# *catena*-Poly[[silver(I)-μ-2-[(pyrazin-2-yl-κ^2^ *N* ^1^:*N* ^4^)amino­meth­yl]phenol] nitrate]

**DOI:** 10.1107/S1600536812034769

**Published:** 2012-08-11

**Authors:** Zhao-Peng Deng, Shan Gao, Seik Weng Ng

**Affiliations:** a Key Laboratory of Functional Inorganic Material Chemistry, Ministry of Education, Heilongjiang University, Harbin 150080, People’s Republic of China; b Department of Chemistry, University of Malaya, 50603 Kuala Lumpur, Malaysia; c Chemistry Department, Faculty of Science, King Abdulaziz University, PO Box 80203 Jeddah, Saudi Arabia

## Abstract

The Ag^I^ atom in the polycationic salt, {[Ag(C_11_H_11_N_3_O)]NO_3_}_*n*_, shows a linear coordination [N—Ag—N = 175.0 (2)°]; the polymeric nature arises from bridging by the pyrazine portion of the ligand, resulting in chains extending parallel to [100]. The NO_3_^−^ counter-ions surround the polymeric chain and inter­act only weakly with it [Ag⋯O = 2.701 (4) and 2.810 (5) Å]. Adjacent chains are linked into a three-dimensional network by O—H⋯O and N—H⋯O hydrogen bonds.

## Related literature

For the structure of 2-{[(pyrazin-2-yl)amino]­meth­yl}phenol, see: Gao & Ng (2012 ▶).
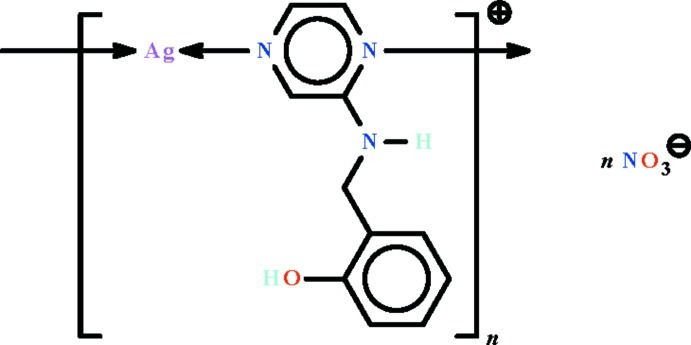


## Experimental

### 

#### Crystal data

[Ag(C_11_H_11_N_3_O)]NO_3_
*M* *_r_* = 371.11
Monoclinic, 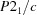

*a* = 7.1265 (9) Å
*b* = 9.5249 (14) Å
*c* = 18.654 (2) Å
β = 97.240 (4)°
*V* = 1256.1 (3) Å^3^
*Z* = 4
Mo *K*α radiation
μ = 1.63 mm^−1^
*T* = 293 K
0.27 × 0.18 × 0.13 mm

#### Data collection

Rigaku R-AXIS RAPID IP diffractometer
Absorption correction: multi-scan (*ABSCOR*; Higashi, 1995 ▶) *T*_min_ = 0.668, *T*_max_ = 0.817
11969 measured reflections
2864 independent reflections
2038 reflections with *I* > 2σ(*I*)
*R*_int_ = 0.042

#### Refinement

*R*[*F* ^2^ > 2σ(*F* ^2^)] = 0.043
*wR*(*F* ^2^) = 0.125
*S* = 1.02
2864 reflections
182 parameters
H-atom parameters constrained
Δρ_max_ = 0.64 e Å^−3^
Δρ_min_ = −0.77 e Å^−3^

### 

Data collection: *RAPID-AUTO* (Rigaku, 1998 ▶); cell refinement: *RAPID-AUTO*; data reduction: *CrystalClear* (Rigaku/MSC, 2002 ▶); program(s) used to solve structure: *SHELXS97* (Sheldrick, 2008 ▶); program(s) used to refine structure: *SHELXL97* (Sheldrick, 2008 ▶); molecular graphics: *X-SEED* (Barbour, 2001 ▶); software used to prepare material for publication: *publCIF* (Westrip, 2010 ▶).

## Supplementary Material

Crystal structure: contains datablock(s) global, I. DOI: 10.1107/S1600536812034769/xu5602sup1.cif

Structure factors: contains datablock(s) I. DOI: 10.1107/S1600536812034769/xu5602Isup2.hkl

Additional supplementary materials:  crystallographic information; 3D view; checkCIF report

## Figures and Tables

**Table 1:** Selected bond lengths (Å)

Ag1—N1	2.172 (4)
Ag1—N2^i^	2.195 (4)

Symmetry code: (i) 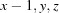.

**Table 2:** Hydrogen-bond geometry (Å, °)

*D*—H⋯*A*	*D*—H	H⋯*A*	*D*⋯*A*	*D*—H⋯*A*
O1—H1⋯O3^ii^	0.84	1.96	2.795 (6)	171
N3—H3⋯O4^iii^	0.88	2.22	2.982 (6)	145

Symmetry codes: (ii) 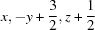; (iii) 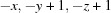.

**Fig. 1.**
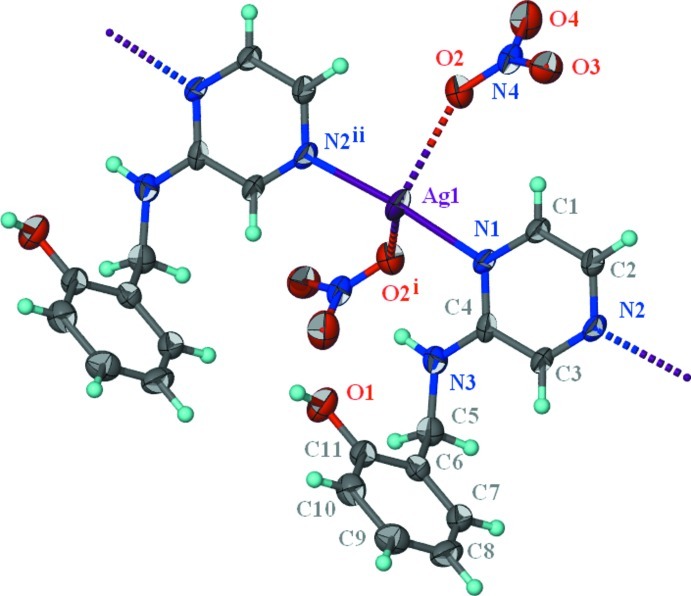
Thermal ellipsoid plot (Barbour, 2001) of the polymeric chain structure of [Ag(C11H11N3O)]nNO3 at the 50% probability level; hydrogen atoms are drawn as spheres of arbitrary radius.

## supplementary crystallographic information

### Comment

A recent study reports 2-[(pyrazin-2-ylamino)methyl]phenol, a reduced Schiff-base that possesses an acidic phenolic group (Gao & Ng, 2012). The reaction with silver nitrate yields polycationic [Ag(C_11_H_11_N_3_O)]*_n_ n*NO_3_ (Scheme I). The polymeric nature arises from bridging by the pyrazine portion of the ligand. The counterions surround the chain and interact only weakly with it [Ag···O 2.701 (4), 2.810 (5) Å] (Fig. 1). Adjacent chains are linked into a three-dimensional network by O–H···O and N–H···O hydrogen bonds (Table 1).

### Experimental

An acetonitrile solution (10 ml) of silver nitrate (1 mmol) was added to a methanol solution (5 ml) of 2-[(pyrazin-2-ylamino)methyl]phenol (1 mmol). The solution was filtered and then side aside, away from light, for the growth of crystals. Colorless crystals were obtained after several days.

### Refinement

Hydrogen atoms were placed in calculated positions (C–H 0.93–0.97, N–H 0.88, O–H 0.84 Å) and were included in the refinement in the riding model approximation, with *U*(H) set to 1.2–1.5*U*(C,*N*,*O*).

### Crystal data

**Table:**

[Ag(C_11_H_11_N_3_O)]NO_3_	*F*(000) = 736
*M**_r_* = 371.11	*D*_x_ = 1.962 Mg m^−^^3^
Monoclinic, *P*2_1_/*c*	Mo *K*α radiation, λ = 0.71073 Å
Hall symbol: -P 2ybc	Cell parameters from 5867 reflections
*a* = 7.1265 (9) Å	θ = 3.1–27.5°
*b* = 9.5249 (14) Å	µ = 1.63 mm^−^^1^
*c* = 18.654 (2) Å	*T* = 293 K
β = 97.240 (4)°	Prism, colorless
*V* = 1256.1 (3) Å^3^	0.27 × 0.18 × 0.13 mm
*Z* = 4	

### Data collection

**Table:**

Rigaku R-AXIS RAPID IP diffractometer	2864 independent reflections
Radiation source: fine-focus sealed tube	2038 reflections with *I* > 2σ(*I*)
Graphite monochromator	*R*_int_ = 0.042
ω scan	θ_max_ = 27.5°, θ_min_ = 3.1°
Absorption correction: multi-scan (*ABSCOR*; Higashi, 1995)	*h* = −9→8
*T*_min_ = 0.668, *T*_max_ = 0.817	*k* = −12→12
11969 measured reflections	*l* = −24→24

### Refinement

**Table:**

Refinement on *F*^2^	Primary atom site location: structure-invariant direct methods
Least-squares matrix: full	Secondary atom site location: difference Fourier map
*R*[*F*^2^ > 2σ(*F*^2^)] = 0.043	Hydrogen site location: inferred from neighbouring sites
*wR*(*F*^2^) = 0.125	H-atom parameters constrained
*S* = 1.02	*w* = 1/[σ^2^(*F*_o_^2^) + (0.0508*P*)^2^ + 4.1394*P*] where *P* = (*F*_o_^2^ + 2*F*_c_^2^)/3
2864 reflections	(Δ/σ)_max_ = 0.001
182 parameters	Δρ_max_ = 0.64 e Å^−^^3^
0 restraints	Δρ_min_ = −0.77 e Å^−^^3^

### Fractional atomic coordinates and isotropic or equivalent isotropic displacement parameters (Å^2^)

**Table:**

	*x*	*y*	*z*	*U*_iso_*/*U*_eq_	
Ag1	0.06666 (5)	0.59659 (5)	0.58246 (2)	0.05110 (18)	
O1	0.3023 (5)	0.4649 (5)	0.8136 (2)	0.0571 (10)	
H1	0.2582	0.5291	0.8375	0.086*	
O2	−0.0170 (6)	0.6917 (5)	0.4458 (2)	0.0575 (11)	
O3	0.1837 (5)	0.8322 (5)	0.4064 (2)	0.0574 (11)	
O4	−0.0257 (6)	0.7272 (6)	0.3321 (2)	0.0658 (12)	
N1	0.3716 (5)	0.5848 (4)	0.5842 (2)	0.0316 (8)	
N2	0.7616 (5)	0.5983 (4)	0.5896 (2)	0.0361 (9)	
N3	0.4029 (6)	0.4097 (5)	0.6707 (2)	0.0434 (10)	
H3	0.2785	0.4081	0.6661	0.052*	
N4	0.0450 (6)	0.7509 (5)	0.3943 (2)	0.0414 (10)	
C1	0.4556 (6)	0.6685 (5)	0.5406 (3)	0.0352 (10)	
H1A	0.3804	0.7245	0.5078	0.042*	
C2	0.6473 (7)	0.6755 (5)	0.5421 (3)	0.0397 (11)	
H2	0.6993	0.7341	0.5100	0.048*	
C3	0.6828 (6)	0.5159 (6)	0.6338 (3)	0.0368 (11)	
H3A	0.7599	0.4648	0.6683	0.044*	
C4	0.4845 (6)	0.5027 (5)	0.6304 (2)	0.0328 (10)	
C5	0.5036 (8)	0.3108 (6)	0.7217 (3)	0.0442 (12)	
H5A	0.6001	0.2642	0.6980	0.053*	
H5B	0.4151	0.2395	0.7333	0.053*	
C6	0.5967 (7)	0.3745 (5)	0.7914 (3)	0.0371 (11)	
C7	0.7896 (7)	0.3554 (6)	0.8134 (3)	0.0451 (12)	
H7	0.8625	0.3062	0.7839	0.054*	
C8	0.8733 (8)	0.4086 (6)	0.8783 (3)	0.0510 (14)	
H8	1.0016	0.3941	0.8926	0.061*	
C9	0.7677 (8)	0.4832 (7)	0.9221 (3)	0.0529 (14)	
H9	0.8247	0.5192	0.9658	0.063*	
C10	0.5791 (8)	0.5042 (6)	0.9013 (3)	0.0472 (13)	
H10	0.5082	0.5552	0.9308	0.057*	
C11	0.4926 (7)	0.4498 (6)	0.8363 (3)	0.0404 (11)	

### Atomic displacement parameters (Å^2^)

**Table:**

	*U*^11^	*U*^22^	*U*^33^	*U*^12^	*U*^13^	*U*^23^
Ag1	0.0201 (2)	0.0684 (3)	0.0657 (3)	0.00250 (18)	0.00874 (18)	0.0059 (2)
O1	0.039 (2)	0.071 (3)	0.062 (3)	0.008 (2)	0.0080 (19)	−0.008 (2)
O2	0.050 (2)	0.066 (3)	0.059 (2)	0.000 (2)	0.018 (2)	0.015 (2)
O3	0.039 (2)	0.064 (3)	0.067 (3)	−0.0142 (19)	−0.0030 (19)	0.011 (2)
O4	0.048 (2)	0.102 (4)	0.047 (2)	−0.010 (2)	0.0027 (19)	−0.012 (2)
N1	0.0186 (16)	0.038 (2)	0.038 (2)	0.0005 (15)	0.0023 (15)	0.0005 (17)
N2	0.0183 (16)	0.044 (2)	0.046 (2)	−0.0003 (16)	0.0028 (16)	0.0007 (19)
N3	0.028 (2)	0.059 (3)	0.043 (2)	−0.006 (2)	0.0052 (18)	0.010 (2)
N4	0.028 (2)	0.046 (3)	0.050 (3)	−0.0013 (18)	0.0086 (19)	−0.002 (2)
C1	0.024 (2)	0.035 (3)	0.047 (3)	0.0019 (19)	0.005 (2)	0.004 (2)
C2	0.029 (2)	0.041 (3)	0.050 (3)	−0.002 (2)	0.012 (2)	0.008 (2)
C3	0.022 (2)	0.048 (3)	0.041 (3)	0.005 (2)	0.0058 (19)	0.002 (2)
C4	0.025 (2)	0.041 (3)	0.034 (2)	−0.0041 (19)	0.0101 (18)	−0.007 (2)
C5	0.042 (3)	0.046 (3)	0.045 (3)	0.002 (2)	0.010 (2)	0.003 (2)
C6	0.038 (2)	0.036 (3)	0.039 (3)	−0.001 (2)	0.009 (2)	0.007 (2)
C7	0.038 (3)	0.047 (3)	0.051 (3)	0.005 (2)	0.013 (2)	0.014 (3)
C8	0.035 (3)	0.049 (3)	0.067 (4)	0.002 (2)	−0.002 (3)	0.014 (3)
C9	0.052 (3)	0.053 (4)	0.051 (3)	−0.008 (3)	−0.005 (3)	0.003 (3)
C10	0.052 (3)	0.046 (3)	0.044 (3)	−0.002 (3)	0.010 (3)	−0.006 (2)
C11	0.035 (2)	0.040 (3)	0.047 (3)	0.001 (2)	0.007 (2)	0.005 (2)

### Geometric parameters (Å, º)

**Table:**

Ag1—N1	2.172 (4)	C1—H1A	0.9300
Ag1—N2^i^	2.195 (4)	C2—H2	0.9300
Ag1—O2	2.701 (4)	C3—C4	1.412 (6)
Ag1—O2^ii^	2.810 (5)	C3—H3A	0.9300
O1—C11	1.376 (6)	C5—C6	1.511 (7)
O1—H1	0.8400	C5—H5A	0.9700
O2—N4	1.242 (6)	C5—H5B	0.9700
O3—N4	1.254 (6)	C6—C7	1.396 (7)
O4—N4	1.225 (6)	C6—C11	1.386 (7)
N1—C1	1.333 (6)	C7—C8	1.377 (8)
N1—C4	1.352 (6)	C7—H7	0.9300
N2—C3	1.314 (6)	C8—C9	1.376 (9)
N2—C2	1.344 (6)	C8—H8	0.9300
N2—Ag1^iii^	2.195 (4)	C9—C10	1.366 (8)
N3—C4	1.342 (6)	C9—H9	0.9300
N3—C5	1.461 (7)	C10—C11	1.390 (8)
N3—H3	0.8800	C10—H10	0.9300
C1—C2	1.364 (6)		
			
N1—Ag1—N2^i^	175.01 (15)	C4—C3—H3A	119.0
N1—Ag1—O2	97.63 (13)	N1—C4—N3	118.3 (4)
N2^i^—Ag1—O2	87.27 (14)	N1—C4—C3	119.3 (4)
N1—Ag1—O2^ii^	93.12 (13)	N3—C4—C3	122.4 (5)
N2^i^—Ag1—O2^ii^	85.21 (14)	N3—C5—C6	115.3 (4)
O2—Ag1—O2^ii^	98.24 (12)	N3—C5—H5A	108.5
C11—O1—H1	109.5	C6—C5—H5A	108.5
N4—O2—Ag1	145.6 (3)	N3—C5—H5B	108.5
C1—N1—C4	117.2 (4)	C6—C5—H5B	108.5
C1—N1—Ag1	119.1 (3)	H5A—C5—H5B	107.5
C4—N1—Ag1	123.6 (3)	C7—C6—C11	118.1 (5)
C3—N2—C2	117.9 (4)	C7—C6—C5	120.7 (5)
C3—N2—Ag1^iii^	122.6 (3)	C11—C6—C5	121.2 (5)
C2—N2—Ag1^iii^	119.1 (3)	C6—C7—C8	120.8 (5)
C4—N3—C5	125.3 (4)	C6—C7—H7	119.6
C4—N3—H3	117.3	C8—C7—H7	119.6
C5—N3—H3	117.3	C9—C8—C7	120.2 (5)
O4—N4—O2	120.3 (5)	C9—C8—H8	119.9
O4—N4—O3	120.3 (5)	C7—C8—H8	119.9
O2—N4—O3	119.4 (5)	C8—C9—C10	120.0 (5)
N1—C1—C2	122.9 (5)	C8—C9—H9	120.0
N1—C1—H1A	118.6	C10—C9—H9	120.0
C2—C1—H1A	118.6	C9—C10—C11	120.3 (5)
C1—C2—N2	120.6 (4)	C9—C10—H10	119.8
C1—C2—H2	119.7	C11—C10—H10	119.8
N2—C2—H2	119.7	C6—C11—C10	120.5 (5)
N2—C3—C4	121.9 (4)	C6—C11—O1	116.7 (5)
N2—C3—H3A	119.0	C10—C11—O1	122.7 (5)
			
N1—Ag1—O2—N4	−19.9 (6)	C5—N3—C4—N1	178.2 (5)
N2^i^—Ag1—O2—N4	161.0 (6)	C5—N3—C4—C3	−0.7 (8)
O2^ii^—Ag1—O2—N4	−114.2 (6)	N2—C3—C4—N1	−5.1 (7)
O2—Ag1—N1—C1	26.1 (4)	N2—C3—C4—N3	173.8 (5)
O2^ii^—Ag1—N1—C1	124.9 (4)	C4—N3—C5—C6	73.9 (6)
O2^ii^—Ag1—N1—C4	−59.3 (4)	N3—C5—C6—C7	−124.6 (5)
Ag1—O2—N4—O4	152.0 (5)	N3—C5—C6—C11	57.3 (6)
Ag1—O2—N4—O3	−26.1 (9)	C11—C6—C7—C8	0.7 (8)
C4—N1—C1—C2	−1.2 (7)	C5—C6—C7—C8	−177.4 (5)
Ag1—N1—C1—C2	174.9 (4)	C6—C7—C8—C9	−0.8 (8)
N1—C1—C2—N2	−1.3 (8)	C7—C8—C9—C10	0.2 (9)
C3—N2—C2—C1	0.5 (7)	C8—C9—C10—C11	0.5 (9)
Ag1^iii^—N2—C2—C1	173.7 (4)	C7—C6—C11—C10	−0.1 (8)
C2—N2—C3—C4	2.6 (7)	C5—C6—C11—C10	178.1 (5)
Ag1^iii^—N2—C3—C4	−170.3 (4)	C7—C6—C11—O1	−179.0 (5)
C1—N1—C4—N3	−174.8 (4)	C5—C6—C11—O1	−0.9 (7)
Ag1—N1—C4—N3	9.2 (6)	C9—C10—C11—C6	−0.6 (8)
C1—N1—C4—C3	4.2 (7)	C9—C10—C11—O1	178.4 (5)
Ag1—N1—C4—C3	−171.8 (3)		

Symmetry codes: (i) *x*−1, *y*, *z*; (ii) −*x*, −*y*+1, −*z*+1; (iii) *x*+1, *y*, *z*.

### Hydrogen-bond geometry (Å, º)

**Table:**

*D*—H···*A*	*D*—H	H···*A*	*D*···*A*	*D*—H···*A*
O1—H1···O3^iv^	0.84	1.96	2.795 (6)	171
N3—H3···O4^ii^	0.88	2.22	2.982 (6)	145

Symmetry codes: (ii) −*x*, −*y*+1, −*z*+1; (iv) *x*, −*y*+3/2, *z*+1/2.
